# Swarming and complex pattern formation in *Paenibacillus vortex *studied by imaging and tracking cells

**DOI:** 10.1186/1471-2180-8-36

**Published:** 2008-02-25

**Authors:** Colin J Ingham, Eshel Ben Jacob

**Affiliations:** 1 Department of Medical Microbiology and Infection Control, Jeroen Bosch Hospital,'s-Hertogenbosh, The Netherlands; 2 Laboratory of Microbiology, Wageningen University, Wageningen, the Netherlands; 3 School of Physics and Astronomy, Raymond & Beverly Sackler Faculty of Exact Sciences, Tel Aviv University, 69978 Tel Aviv, Israel

## Abstract

**Background:**

Swarming motility allows microorganisms to move rapidly over surfaces. The Gram-positive bacterium *Paenibacillus vortex *exhibits advanced cooperative motility on agar plates resulting in intricate colonial patterns with geometries that are highly sensitive to the environment. The cellular mechanisms that underpin the complex multicellular organization of such a simple organism are not well understood.

**Results:**

Swarming by *P. vortex *was studied by real-time light microscopy, by *in situ *scanning electron microscopy and by tracking the spread of antibiotic-resistant cells within antibiotic-sensitive colonies. When swarming, *P. vortex *was found to be peritrichously flagellated. Swarming by the curved cells of *P. vortex *occurred on an extremely wide range of media and agar concentrations (0.3 to 2.2% w/v). At high agar concentrations (> 1% w/v) rotating colonies formed that could be detached from the main mass of cells by withdrawal of cells into the latter. On lower percentage agars, cells moved in an extended network composed of interconnected "snakes" with short-term collision avoidance and sensitivity to extracts from swarming cells. *P. vortex *formed single Petri dish-wide "supercolonies" with a colony-wide exchange of motile cells. Swarming cells were coupled by rapidly forming, reversible and non-rigid connections to form a loose raft, apparently connected *via *flagella. Inhibitors of swarming (*p*-Nitrophenylglycerol and Congo Red) were identified. Mitomycin C was used to trigger filamentation without inhibiting growth or swarming; this facilitated dissection of the detail of swarming. Mitomycin C treatment resulted in malcoordinated swarming and abortive side branch formation and a strong tendency by a subpopulation of the cells to form minimal rotating aggregates of only a few cells.

**Conclusion:**

*P. vortex *creates complex macroscopic colonies within which there is considerable reflux and movement and interaction of cells. Cell shape, flagellation, the aversion of cell masses to fuse and temporary connections between proximate cells to form rafts were all features of the swarming and rotation of cell aggregates. Vigorous vortex formation was social, i.e. required > 1 cell. This is the first detailed examination of the swarming behaviour of this bacterium at the cellular level.

## Background

Bacteria cultured on semi-solid media display colony morphologies which are characteristic of a species, strain and/or particular mode of growth [1-19]. Colony recognition is a major feature of microbiology, with applications in species identification and phenotyping. The structure of cells and the extracellular material within colonies and biofilms is generated by the interplay of many factors including gradients of nutrients and waste products, extracellular polymers and surfactants, cell surface properties, programmed cell death and differentiation, environmental sensing and intercellular signaling [19-41]. In some instances, the resultant masses of cells are striking, with intricate patterns of growth that cover the surface of a Petri dish from a single point of inoculation. Pattern formation is an intriguing form of multicellular organization of the microbial community [42-48]. Microbial pattern formation has been used as a model for complex open systems and as the basis for understanding the organization of cells in eukaryotic tissues and diseases [43,49,50].

Bacterial motility is a dispersal mechanism involved in generating macroscopic patterns. For example, the periodic flagella-driven swarming of the Gram-negative bacterium *Proteus mirabilis *results in concentric rings of alternating swarming and vegetative growth [51]. *Myxococcus xanthus*, another Gram-negative colonial bacterium but one with gliding motility, generates fruiting bodies in which different cells make unequal contributions; some cells effectively sacrifice themselves for the colony [31,32,34]. The microaerophilic bacterium *Thiovulum majus *can form complex honeycomb patterns on marine surfaces [52]. Gram-positive bacteria, related to *Bacillus subtilis*, use flagella to swarm to form elaborate and convoluted colonies with chiral and/or fractal geometries. Lubricant polymers and detergents are involved in many of these processes [53,54]. However, the biological basis of pattern formation, particularly in Gram-positive bacteria, is not well understood.

Not all dispersal in extended bacterial colonies is generated by motility. *B. subtilis *can form complex multicellular structures without motility, such as macrofibres on 2D surfaces or fruiting bodies [55]. *Bacillus mycoides *is also able to create asymmetric patterns from growth of extended chains of non-motile but filamentous cells [19]. Non-motile strains of the *Enterobacteriaceae *can also form fractal patterns [21]. As with motile organisms, surfactants and extracellular polymers are often important in facilitating colony extension [19,21].

Some *Paenibacillus *species (Gram-positive, spore-forming aerobes that were previously classified as *Bacillus *species), can exhibit complex pattern forming behavior. Clinical isolates of *P. alvei *swarm over agar plates [56]. *Paenibacillus *spp., such as *P. alvei *and *P. denditiformis*, have different repertoires of patterns or morphotypes [4,5,8,9,16,39,45-48,57]. *P. vortex *is a motile and colonial *Paenibacillus *which swarms so effectively on agar that it is difficult to maintain it as an isolated colony. *P. vortex *is so named because of the vigorous rotation of entire colonies, and of areas within colonies [14,16,26]. As for other swarming Gram-positive bacteria, development of social motility in *P. vortex *is dependent on the properties of the surface being traversed, including the agar concentration and the degree to which it is lubricated by the bacterium [39,45-48]. Swarming is exceptionally sensitive to the environmental conditions, including nutrients, humidity and stresses such as those imposed by sub-lethal doses of antibiotics [35]. One particular property of *P. vortex *is that some of the information required to form features of a pattern is apparently retained through successive generations [39,46-48]. *P. vortex *is not well developed with respect to genetics or molecular biology.

The recent isolation of *P. vortex *may offer a significant advantage in studying complex social organization. Many of the multicellular characteristics of bacteria are lost with serial sub-culturing and so can be deficient in the model laboratory strains. For example, biofilm development, fruiting body formation and swarming are all found more robustly in less passaged isolates of *B. subtilis *[53] than in the "highly domesticated" strain *B. subtilis *168. Also, a *Bacillus mycoides *type strain was found to have lost the ability to organize into complex patterns and recent work on this phenomenon was necessarily performed on fresh isolates [19]. *M. xanthus *complex multicellular properties are also selected against by prolonged growth in a homogeneous environment [27]. *P. vortex *was originally isolated as an outgrowth from a *B. subtilis *colony. Isolation of pattern-forming bacteria from *B. subtilis *cultures appears to have occurred several times [4,6,57-59].

The fascinating subject of microbial pattern formation remains to be explored in detail by microbiologists. In this work we describe this phenomenon in *P. vortex *grown mainly on rich agar plates. The complex bacterial dynamics and organization were monitored by *in situ *electron microscopy; moving cells were imaged in real time by light microscopy and the spread of cells within colonies was mapped by tracking mutants.

## Results

### Macroscopic pattern formation by *P. vortex *on gel surfaces

**Figure 1:**
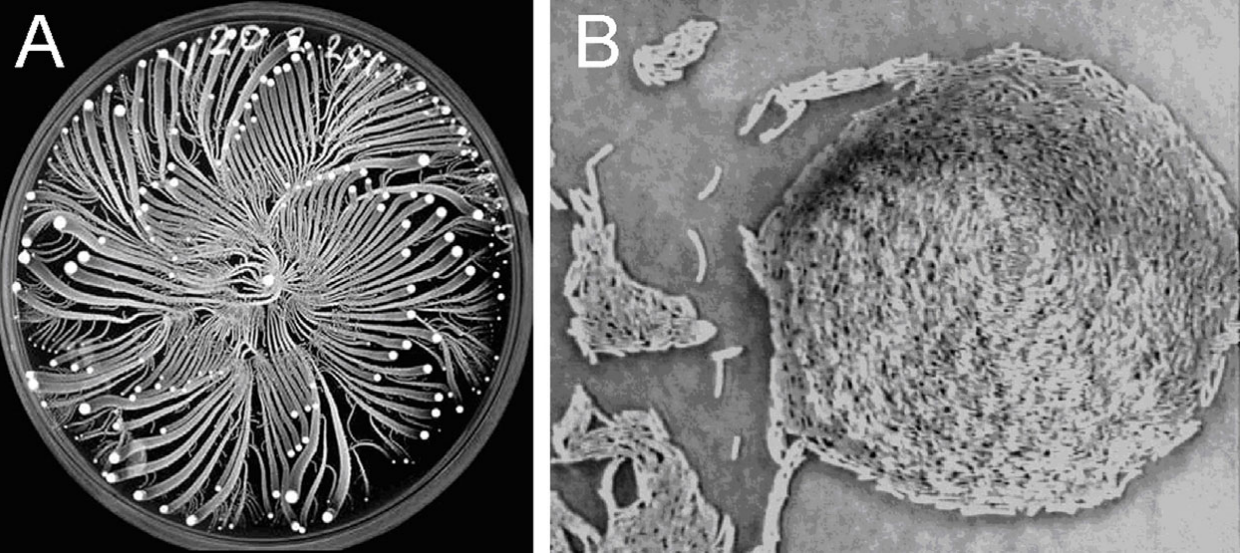
**A typical colonial pattern generated by *P. vortex *when grown on 2.25% (w/v) agar with 2% (w/v) peptone in a 9 cm Petri dish**. **A. **Whole colony view. Each vortex (the condensed group of bacteria – the bright dots) is composed of many cells that swarm collectively around their common center at about 10 μm/s. **B. **An individual vortex – the diameter of the vortex is 75 μm. Both clockwise and anticlockwise rotating vortices are observed, although the majority have the same handedness.

**Figure 2:**
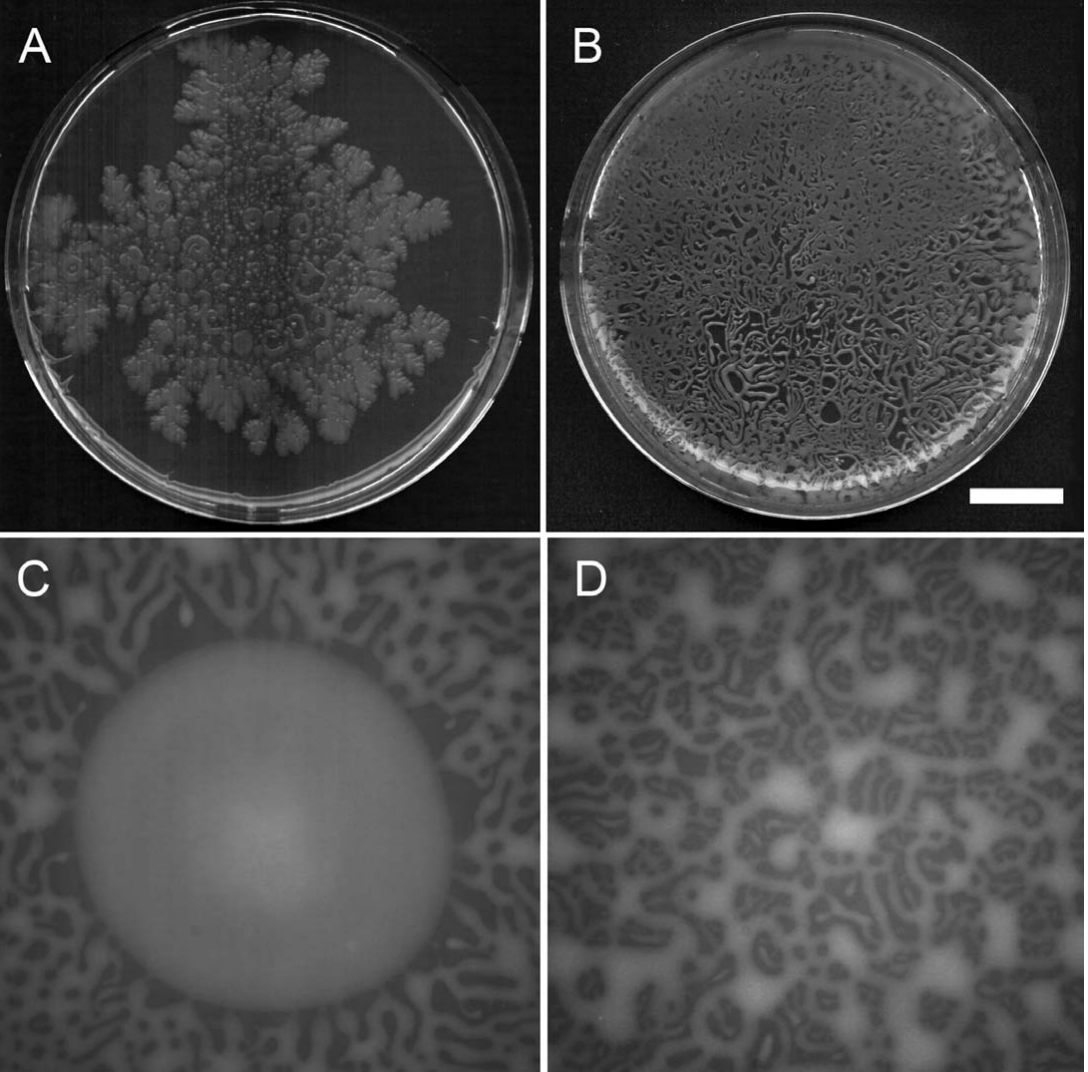
**Macroscopic pattern formation by *P. vortex *on MH agars**. In all pictures the lighter areas are interconnected (moving) masses of cells and darker areas are uncolonized agar; all pictures taken after 18 h from Petri-dish-wide colonies. **A. **Swarming on 1.5% (w/v) agar MH plates from a point inoculation showing fractal pattern and (centrally) detached colonies. **B. **Swarming on 0.3% (w/v) agar MH plates after 28 h in which a network forms across the plate then elaborates. **C. **Detail of inoculation from a droplet on 0.3% agar with outgrowth at specific points from the central circular mass. **D. **Detail of pattern 30 mm from the center from the same plate as (C). Scale bar in (B) represents 2 cm when applied to panels (A) or (B) and 2 mm for (C) or (D).

Previously, colonial development of the *P. vortex *bacteria was studied during growth on Peptone plates and relatively hard surfaces (≥ 2% w/v agar). An example of representative colonial pattern in such setting is shown in Fig 1A. For more details see Refs [14,16,26,30,45-48]. These patterns are characterized by the formation of vortices – aggregates of tens to thousands of cells that rotate around a common center and move as a coherent unit. On softer surfaces (not shown here) the colonies are no longer composed of vortices and their shapes ("snakelike") was similar to that exhibited in Fig. 2A during growth on Mueller-Hinton (MH) plates.

The *P. vortex *cells in the vortices that develop on plates with high concentrations of agar replicate, and the vortex expands in size and moves outward as a unit, leaving behind a trail of motile but usually non-replicating cells – the vortex branch. The vortices vary in size from tens to thousands of bacteria, according to their location in the colony (Fig. 1B). The dynamics of the vortices is quite complicated and includes attraction, repulsion, merging and splitting of vortices. However, these patterns take a relatively long time to form (days). Additionally, motility on very high concentrations of agar tend not to be sustained with illumination during imaging. Because of these two factors, the study was performed on MH agar at < 0.7% and 1.5% w/v agar (termed low and high percentage agar respectively) upon which motility was vigorous and sustained, which suited the dynamic imaging and tracking approach used.

Inoculation of MH agar plates resulted in the initiation of swarming motility within 5–8 h when incubated at temperatures from 25 to 37°C. Swarming occurred on the surface of agar at concentrations from 0.3 to 2.2% (w/v) but only under aerobic conditions; no growth was observed under anaerobic conditions. At 2.2 to 2.6% (w/v) agar swarming occurred but was erratic, i.e. not always initiating and sometimes stalling without covering the entire plate. The patterns of swarming on 1.5% MH agar showed fractal patterns (Fig. 2A) or and/or dispersed colonies similar to those previously described during growth on Peptone plates [14,16,26,30,45-48]. At lower agar concentrations swarming was a highly efficient method of dispersal and growth. In liquid culture, aerated with vigorous shaking overnight, 20 ml of MH medium supported 1.3 × 10^9 ^cfu/ml. Swarming solely on the surface of 20 ml of MH (0.3% w/v) agar supported up to 7.7 × 10^8 ^cfu/ml. Colonial growth, but not swarming, was observed at 42°C irrespective of the agar concentration. Where multiple inoculations were made on the same plate, colonies fused provided the cells were swarming rapidly, > 3 mm/h (data not shown). Such collisions occurred on both high and low percentage agars. However, where swarming was relatively slow (< 3 mm/h) then colonies did not merge, which is as previously described for swarming on peptone-based media [14,16,26,30,45-48].

At low agar concentrations (< 0.7 %) detachment of aggregates was never observed; all regions of microbial growth were visibly a single network. The arrangement of the spreading colony was fairly evenly spaced masses of > 4 mm across linked by 2–4 connecting strands of < 0.7 mm and spaced every 4–8 mm or a more even network (Fig. 2B). It was possible to culture highly branched, macroscopic colonies over 8 cm across. The patterns formed suggested that whilst motile masses of cells could meet, as indicated by the formation of rings and loops, there was a degree of avoidance of this event. This is implied by the fact that in the later stages of swarming there was a tendency for multiple outgrowths to invade the remaining open areas of agar but not fuse with other cell masses (Figs. 2C and 2D).

### Real time imaging of swarming motility

#### Low percentage agars

**Figure 3:**
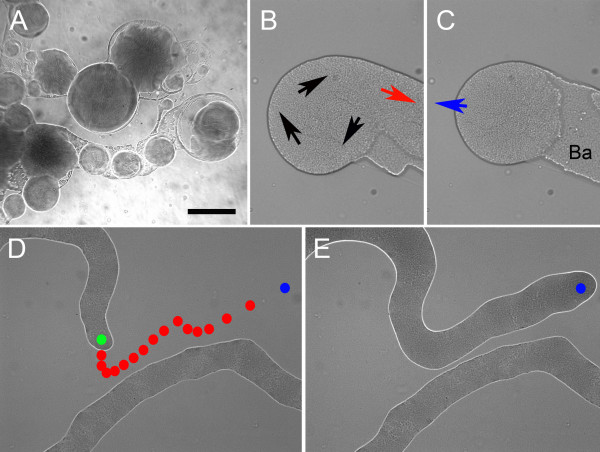
**Dynamic imaging of swarming by light microscopy**. Stills from movies of swarming motile masses of *P. vortex*. **A. **Rotating colonies spinning out from a central mass on MH (1.5% w/v) agar. Material between rotating colonies (all are in motion) is highly reflective basal material with a small number of cells. **B. **Start of detachment of rotating colony from the edge of a swarming culture on MH (1.5% w/v) agar. Black arrows indicate direction of rotation, with the bulk of cells withdrawing (red arrow) to leave colony isolated. **C. **As (B), 5 min later with further withdrawal of main mass of cells; a few minutes after this the spinning colony moved away in the direction of the blue arrow. Ba = reflective basal material. **D. **Moving mass of cells on MH (0.3 % w/v) agar; first image with the center of the tip shown as a green dot and the track of subsequent movement shown as the position of the tip one red dot every 20 s. The tip of a moving mass avoids a second moving strand of cells to which it is interconnected. **E. **From the same image stack as D but taken 10 min later with same (last) position shown in both (D) and (E) as a blue dot.

On agars < 0.7 % (w/v) motility was vigorous, with extension rates of cell masses in excess of 1 cm/h under optimal conditions. Detachment of cell masses into isolated colonies was never seen. The motile, extending "snakes" typical of this environment showed a marked aversion to fusing with other cell masses. This was the case for 6/6 studies of masses within 40 μm of each other over 2–3 h; in all cases the "snake" changed direction, apparently to avoid the collision. Fig. 3D shows a typical example, where the tip of the snake veered away from another cell mass (in this case a section of the same elongating cell mass) twice within 2 h. We were unable to catch any fusion events between cell masses in real time over a period of a few hours.

Dilution of motile masses (c. 10-fold), using drops of pre-warmed MH medium, resulted in visible motility of > 95% of the cells in the field of view. Subsequently, rafts of cells reformed (within 2–15 min) in which the cells were still motile but were noticeably more aggregated. Observation of cells swimming immediately after greater dilutions suggested that cells in liquid culture swam at speeds up to 10 μm/s, provided there was access to oxygen. The organism appeared positively aerotactic: cells accumulated around air bubbles when motile but rapidly lost motility under a cover slip in the absence of such bubbles. Motility in a liquid environment was only seen with cells diluted from swarm plates and was not observed in MH liquid culture.

#### High percentage agars

At concentrations of agar above 1% (w/v), aggregates of hundreds to thousands of cells were able to detach from the central mass of cells to form rotating groups (vortices) or were part of the same interconnected pattern (Fig. 2A, Fig. 3A). Movement rates for entire colonies could be in excess of 1 cm/h with a rotation rate of up to once every few seconds. Within the mass of cells distinct whirlpool patterns and streams were observed. The mechanism of detachment of entire sub-colonies in this situation appeared to be primarily withdrawal of cells into the central mass and not simply the colony pulling away. The position of the colony was essentially static during detachment until the connection was broken with the central body. Detachment of a sub-colony was preceded by a mass of cells flowing back into the main colony body (Fig. 3B and 3C).

### Effects of extracellular material on motility and pattern formation

**Figure 4:**
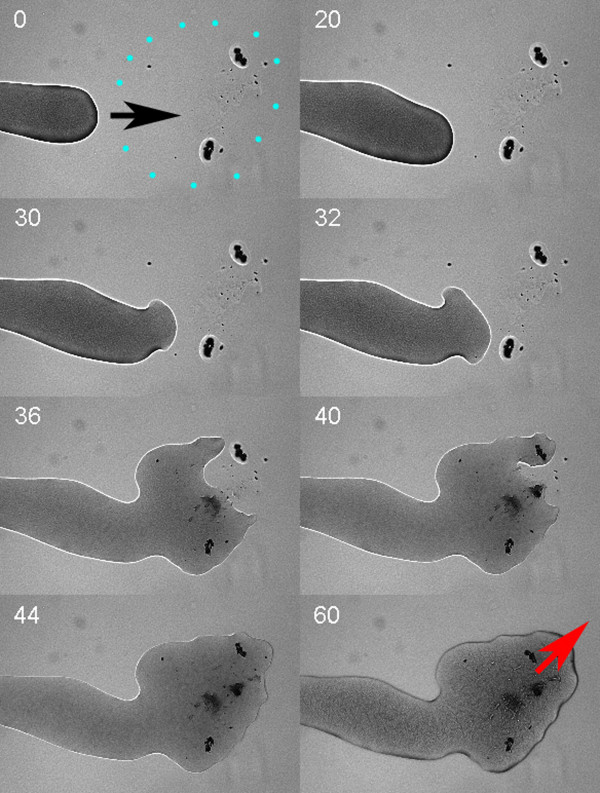
**Effect of extracellular material from plates containing swarming cells on swarming**. Light microscopy of *P. vortex *moving on MH agar (0.3% w/v), extending into an area where extracellular material derived from washes of swarming cells was delivered by toothpick and allowed to soak into the agar. Time of frame capture is noted in seconds. **Second 0**: Area of extract outlined in blue dots with direction of cell mass elongation shown by the black arrow. Dark marks inside the area of the extract are disturbances due to the toothpick contacting the agar. **Second 30**: cell mass starts to disperse as it contacts the area of the extract. **Second 44**: Cell mass has dispersed into area of extract. **Second 60**: Additional cells are moving into this area from further back in the colony; the cell mass is growing in volume at the tip and extends in the direction of the red arrow.

Preparations of the extracellular material from swarming *P. vortex *cells contained one or more activities that affected the motility of entire masses of cells. When a droplet (10 μl) was delivered ahead of an extending "snake" body of cells the result was a temporary dispersal of the cells into the area of the extract (Fig. 4). It was not clear if the effect was chemotactic or a physical effect, such as detergent activity. Control experiments (not shown) demonstrated that toothpick marks in the absence of cell extract or growth medium did not have the same effect. When present at < 5% (v/v) in MH agar (1.5% v/v) the extracellular material had little obvious effect on pattern formation (data not shown). When tested for detergent activity, using the drop spreading method, cell extracts had a greater detergent activity than MH medium alone.

### Individual cell morphology and flagellation during swarming

**Figure 5:**
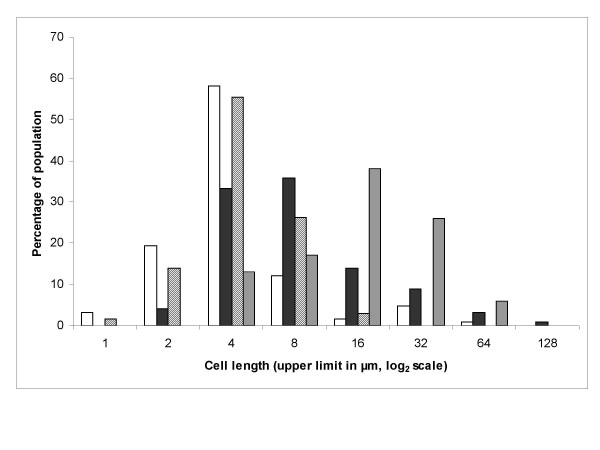
**Analysis of cell length**. Cell lengths (n = 200) were measured from digital images of *P. vortex *cultures. White bars: swarming on 1.5% MH agar at 37°C. Black, swarming on 1.5% (w/v) MH agar at 37°C with MitC (0.3 μg/ml). Diagonal hatching, non-motile culture on 1.5% (w/v) MH agar at 37°C with Congo Red (400 μg/ml). Horizontal hatching, culture on 1.5% (w/v) MH agar at 42°C.

Measurements of individual cells indicated that *P. vortex *is distinctly curved (average 18.6° deviation from a straight line) compared to *B. subtilis *168 (7.1°). The bend in *P. vortex *was significantly different to *B. subtilis *168 by Student's *T*-test (P > 0.001, n = 200 for both samples). The most extreme deviation from a linear cell in *P. vortex *seen was 101° (i.e. an acute angle). In contrast, the maximum bend was only 19° for cells of *B. subtilis *168. Measurements of cell length by light microscopy and analysis of digital images (Fig. 5) suggested that there was a subpopulation of extremely elongated cells to be found within the swarming population. The elongated subpopulation was often a minority, at 37°C around 5% of swarming cells were above 16 μm long. Cell length also increased dramatically with growth on agar at 42°C, in this situation > 33% of the population was above 16 μm long. In contrast, elongated cells above 16 μm were not usually seen in liquid culture but rather appeared to be a feature of growth on a semi-solid medium.

#### High percentage agars

**Figure 6:**
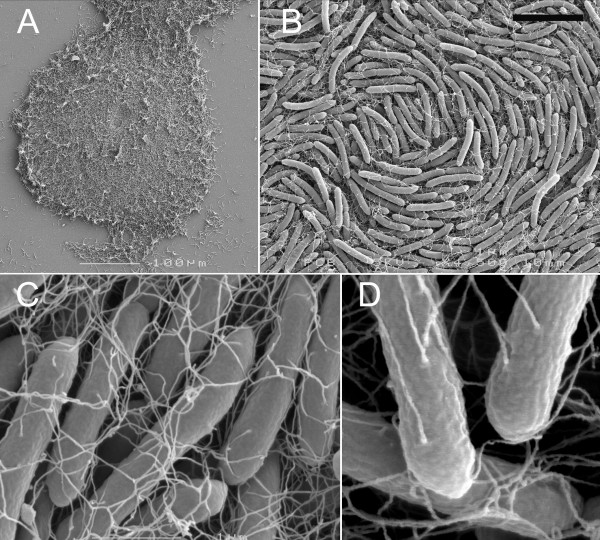
**SEM of actively swarming *P. vortex *cultured on 1.5% (w/v) agar MH plates**. **A. **Overview of small rotating colony which is contacting another moving and similar-sized colony (top) and extending a short process that is likely to be the start of a third mass (bottom). **B. **Center of colony with typical vortex pattern. **C. **Swarming *P. vortex *showing entangled flagella. **D. **Attachment to cells of filaments presumed to be flagella. Scale bar in (b) 5 μm, 1 μm when applied to (C) and 400 nm in (D).

**Figure 7:**
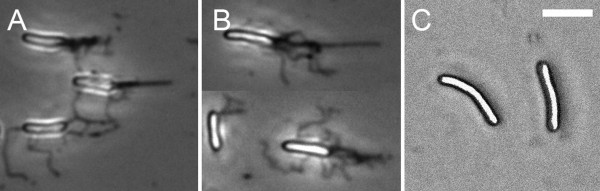
**Flagella staining of *P. vortex***. Cells from cultures stained for flagella were viewed by transmission light microscopy. **A. **Cells swarming on MH agar (0.3% w/v) showing individual or bundles of flagella as dark threads. **B. **Cells swarming on MH agar (1.5% w/v). **C. **Non-motile cells from liquid culture lacking flagella. Scale bar (top right) is 3 μm.

A rapid fixation method, combined with the use of osmium tetroxide to preserve hydrated polymers during critical-point drying, was used to image swarming bacteria by SEM (Scanning Electron Microscopy). Imaging of rapidly fixed rotating colonies on MH agar (1.5% w/v) was possible using this technique (Fig. 6A). Colonies were flattened with depressions or troughs and peaks, including the periphery of the colony. Most notable in these pictures was the alignment of the curved cells into the vortex or whirlpool pattern (Fig. 6B). The presence of filaments extending from the cell surface was notable and ubiquitous for swarming cells (Fig. 6B–C). These filaments were of dimensions consistent with flagella (< 60 nm wide, > 5 μm long); they showed a strong tendency to be entwined with those of the near neighbors (Fig. 6C) that may indicate an unusual degree of flexibility. Helical bundles, in which peritrichous flagella from the same cell aggregate into a single rotating mass to drive motility of a cell, were never observed in swarming cells [2]. The density of filaments was similar in the elongated subpopulation when grown at 37°C or less, 2 to 8 flagella per μm of cell length. Examination of all positions in motile *P. vortex *swarms gave the same result; the entire colony was covered in a network of these entwined filaments. The filaments resembled the lateral flagella of Aeromonas [60]. As a control, *P. mirabilis *swarmers were fixed and imaged by the same method; here helical flagella bundles were seen that match previous observations [61], which suggests that the fixation method was able to capture the structure of rapidly swarming bacteria. Flagellar staining and light microcopy confirmed that swarming *P. vortex *was peritrichously flagellated with numbers and lengths of flagella consistent with the SEM images (Fig. 7B). Liquid growth led to the loss of both flagella and motility (Fig. 7C). On the basis of these data we conclude that we are viewing bacteria which develop peritrichous or lateral flagella when swarming on a surface.

#### Low percentage agars

Light microscopy of swarming on MH (0.3% w/v) agar lead revealed similar arrangements of flagella and distribution of cell lengths and curvature to that found on higher percentage agars (Fig. 7A).

### Tracking of the dispersal of an antibiotic resistant mutant within macroscopic colonies

*P. vortex *was found to be RIF^S ^(rifampicin sensitive) when tested on MH agar (0.3 to 2% w/v agars) using antibiotic-containing Neo Sensidisks, which produced zones of clearing of c. 12 mm in the bacterial lawn. Spontaneous, RIF^R ^(resistant) colonies of *P. vortex *were obtained at a frequency of 1.7 × 10^-9 ^when selected on MH plates (1.5% w/v agar) containing 50 μg/ml RIF. This was a similar frequency to the occurrence of spontaneous RIF^R ^in *B. subtilis *[62]. A RIF^R ^mutant was selected with wild-type growth and swarming characteristics. Macroscopic colonies, from 0.5 to 9 cm in diameter on MH agar (0.3% w/v), of the wild-type strain were seeded with an actively motile RIF^R ^strain (from another swarm plate with the same percentage of agar) at specific locations. After 1–20 h replica plating was used to sample locations within the wild-type colony for the arrival of representatives of the RIF^R ^strain. Control experiments, replica plating RIF^S ^colonies, indicated that colonies without the RIF^R ^strain did not produce RIF^R ^colonies sufficiently often to confound this method of analysis.

#### Low percentage agars

**Figure 8:**
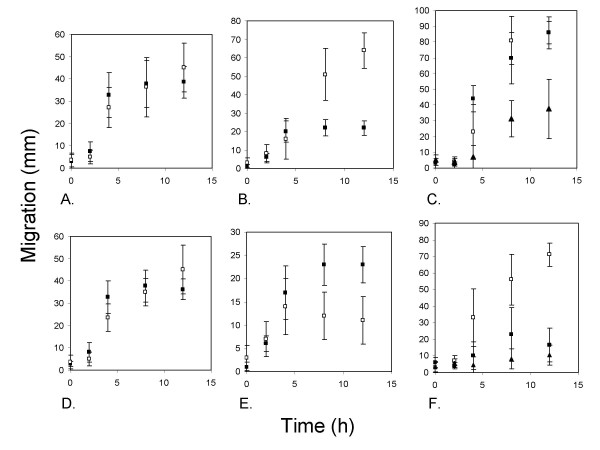
**Dispersal of a RIF^R ^strain of *P. vortex *within extended colonies of the RIF^S ^strain**. All graphs plot the average migration distance of the RIF^R ^strain against time (± SD) on MH agar (0.3% w/v in panels A-C or 1.5% w/v agar in panels D-F) when inoculated into a RIF^S ^colony and incubated at 37°C. **A. **Inoculation of the RIF^R ^strain at the edge of the initial ring, 0.5 mm from center of the dish at the same time as the RIF^S ^strain (■) and 5 h after (□). **B. **Inoculation of actively swarming 30 mm radius RIF^S ^colonies at the outer edge of the colony with the RIF^R ^strain. (■) Outwards migration of RIF^R ^subsequently tracked and limited to 22.5 mm by the edge of the Petri dish. (□) Inwards migration. **C. **Inoculation of Petri-dish-wide RIF^S ^colonies at one edge with RIF^R ^strain. (□) Immediately after swarming RIF^S ^cells reached edge of Petri dish, (■) 12 h later (▲) 36 h later. **D**. As (A) except on1.5% (w/v) agar. **E. **As (B) but on 1.5% (w/v) agar. **F. **As (C) but on 1.5% (w/v) agar.

Rapid spread of the RIF^R ^strain occurred throughout the colony (Fig. 8). The only exception was that inoculations in the absolute center of the colony were less reliable in facilitating migration of the RIF^R ^strain than those made 0.5 mm away from the center (data not shown). Spread was identical whether the resistant strain was inoculated near the center of a plate with the sensitive strain or 5 h after (Fig. 8A). Inoculation of swarming colonies when at a 30 mm radius (i.e. still actively spreading), allowed re-isolation of the RIF^R ^strain at both the periphery and interior of the colony 3–8 h later (Fig. 8B). Both outwards and inwards movement appeared to be occurring with similar efficiency. Inoculation at the periphery of a Petri-dish-wide colony resulted in efficient spread of the RIF^R ^strain across the 9 cm plate within 8 h. Intra-colony migration of cells in this situation occurred at a similar rate to colony extension. Spreading right across the Petri dish was still possible when the inoculation was made 12 h after the colony had reached the edge of the dish (Fig. 8C). Spread was reduced to a few centimeters at most (assaying 12–36 h) from the inoculation point when the inoculation of the RIF^R ^strain was made 24–36 h after the RIF^S ^strain reached the edge of the Petri dish (Fig. 8C). The replica plating method also indicated that the RIF^R ^strain was spreading by most or all available paths, i.e. generally through the complex network formed by the RIF^S ^strain rather than *via *a specific or limited set of routes. These data support the concept that a single, macroscopic colony is formed on low percentage agars with the potential for the efficient exchange of cells between distant locations. These results were supported by transmission microscopy of colonies directly on Petri dishes, which suggested that multidirectional streaming was a general feature of colony dynamics during colony expansion, and up to 12–24 h after the leading edge reached the Petri dish wall.

#### High percentage agars

On higher percentage agars spread of the RIF^R ^strain was also observed and all inoculation points within a Petri-dish-wide colony allowed access to essentially all other locations within a 4–12 h period. However, after this time migration was less effective than on lower percentage agars and the period in which rapid migration of the RIF^R ^strain within the macrocolony less sustained (Fig. 8F). Additionally, there was a greater bias towards outwards movement: once the colony exceeded 30 mm diameter the RIF^R ^inoculation tended to spread in an arc of c. 220–300 degrees, i.e. in most directions except directly inwards (Fig. 6E). As with migration of the RIF^R ^strain on lower percentage agars, regions to which the RIF^R ^strain did spread were completely colonized. These data suggest that exchange of cells between rotating colonies, even when apparently separated, was relatively commonplace.

### Effect of PNPG on swarming motility

Addition of PNPG (*p*-Nitrophenylglycerol, 0.5 mM) to MH plates (1 to 1.5% w/v agar) delayed the initiation of swarming by 4–8 h. In liquid culture this concentration was not inhibitory to growth (doubling time 27 min without PNPG, 29 min with 0.5 mM in MH broth at 37°C). Higher concentrations were inhibitory growth. PNPG at 0.5 mM is sufficient to inhibit *Proteus *swarmers as is used as such within clinical microbiology laboratories to limit this organism in diagnostic assays on agar [63].

### Effect of mitomycin C on cell morphology and swarming motility

Mitomycin C (MitC), a DNA damaging agent and a strong trigger for the SOS response [64,65] was used to create stresses on the swarming cells. When used at concentrations from 0.1 to 0.3 μg/ml MitC did not reduce the growth rate in liquid culture (doubling time 27 min without MitC, 31 min with 0.3 μg/ml in MH broth at 37°C). However, MitC did cause filamentation, as would be expected for any rod-shaped bacteria in which the SOS response is strongly induced [64,65]. Filamentation was also seen on solid media but, additionally, more curved cell morphologies were found, to the extent that loops could be formed from a single cell.

#### Low percentage agars

At the microscopic level on low percentage agars it appeared that unproductive or abortive branches were being formed that failed to extend far (< 100 μm) from the parental body in addition to productively elongating "snakes". The structure of colonial development was more modestly affected, with abortive side branch formation being the most common outcome.

#### High percentage agars

**Figure 9:**
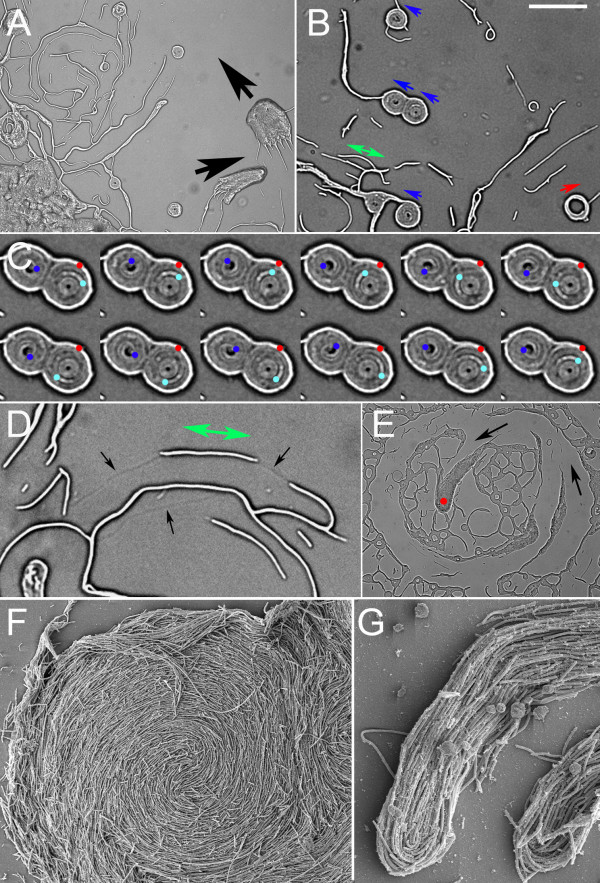
**Effect of MitC on cell morphology and swarming on high percentage agars**. Stills from movies made by transmission light microscopy. **A. **Masses of cells moving in the direction of the black arrows and trailing elongated cells. Small circular foci are rotating (next panels). **B. **Cells grown as in A. Highly coiled rotating bodies of 1–10 cells are marked with the direction of rotation indicated by an arrow. A double-headed arrow indicates that the elongated cell below it was displaying bidirectional motility, shunting slowly (< 5 μm/min) backwards and forwards. Single headed blue and red arrows indicate anticlockwise and clockwise rotation, respectively. Single headed red arrow is clockwise. **C. **Detail of (B) showing rotation in detail (every third frame shown, frames 1 to 18 in top row, 21 to 36 in bottom row reading from left to right) with a rotation rate of once every 3–4 s. Three positions are marked on specific cells. The red dot is a fixed point on an outer (non-rotating) cell whilst the dark and light blue spots track the rotation of two cells in the adjacent foci. All cells in this section are rotating except for the outer layer. **D. **Elongated cell (below the green arrows) displaying slow reversible motion down apparent tracks or imperfections in the agar surface (black arrows). **E. **Movement of masses of cells (red dot) through more scattered cells with the most recent track indicated by arrows. **F. **SEM of colony on 1.5% MH agar with 0.3 μg/ml MitC showing that the vortex pattern of cells is maintained despite elongation (compare Fig. 3). **G. **Tips of extending bodies similar to that viewed by light microscopy in panels (A) and (E). Scale bar in panel (B) indicates; 60 μm when applied to panel (A), 60 μm for (B), 20 μm for (C), 100 μm for (E), 30 μm for (F) and 20 μm for (G).

The effect of MitC on swarming was more extreme on 1.5% (w/v) MH agar. Real-time imaging revealed that even extremely elongated or curved cells were motile in masses (Fig. 9A,B,D,E). Single isolated cells, when highly curved, showed "tail chasing" rotation, albeit slowly (< 0.1 rpm). Elongated cells appeared to sometimes move along tracks in the agar (Fig. 9D). We were not able to determine whether these tracks were being created by the cells or whether the cells were effectively moving and/or elongating to align with preexisting imperfections in the agar surface. Small groups of 3–20 cells could also show a rapid (up to several times a second) and quite extraordinary rotation around a common center resembling a wheel. Examples of two such rotating wheels are shown in Fig. 9C. In this case there are two rotating groups of cells with the outer layer of cells non-motile. Rotating wheels (vortices) were often seen in close contact with non-motile, more linear cells (Fig. 9). Both clockwise and counterclockwise rotation was seen within wider fields of view, but for any given instance the spinning motion was usually unidirectional and without detectable pausing. Larger masses of cells formed in a similar fashion to the rotating colonies seen without MitC but were less coordinated and rarer. Masses often trailed elongated cells (Fig. 9A and 9G) and were generally less rounded than rotating colonies without MitC. These showed no aversion to crossing the paths of groups of < 10 cells and their passage resulted in the alignment of less motile cells. Larger masses of cells (hundreds of microns across) showed a very striking vortex pattern; despite the elongated cells the drive towards rotation of cell masses was strong (Fig. 9F). At the macroscopic (visible to the eye) level swarming morphology became coarser and less highly branched as a result of MitC treatment.

#### Effect of Congo Red on swarming motility

**Figure 10:**
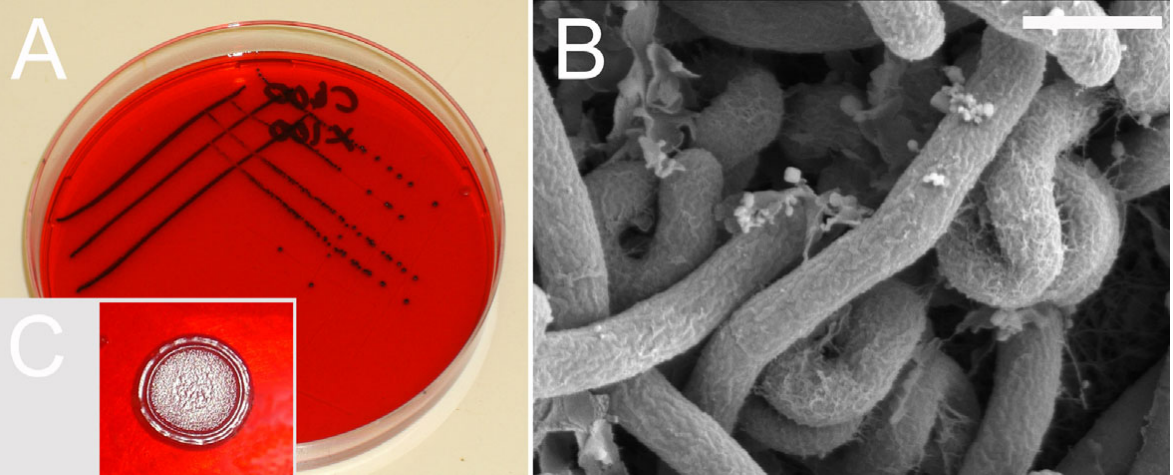
**Effect of Congo Red on Swarming and Cell Morphology**. **A**. *P. vortex *streaked on MH agar (1.5 % w/v) with 400 μg/ml Congo Red and 100 μg/ml X-gal (to add contrast for photography). **B. **SEM of cells cultured with Congo 400 μg/ml Red with frequent convoluted/bent cells and absence of flagella (compare with Fig 3). **C. **Spot plating on Congo Red showing ringed colony but no motility after 48 h. 24 h point the cells in the control plates without Congo Red had reached the edges. Scale bar in panel (B) is 2 cm when applied to (A), 1 μm for (B) and 1 cm for (C).

Addition of Congo Red (100 μg/ml) delayed the initiation of swarming by 6–8 h on solid media (1.5 % w/v agarose). Higher concentrations of Congo Red (400 μg/ml) completely inhibited swarming (> 72 h), but still permitted growth, resulting in round, raised colonies up to 8 mm in diameter with a ringed edge from drop inoculations and conventional colonies from streak plating (Fig. 10A and 10C). Examination of cells grown with Congo Red by light microscopy indicated they were not motile and tannin staining did not reveal flagella when viewed by light microscopy. SEM images of cells showed an absence of the filaments observed in motile cells, and an extreme morphology: highly coiled and twisted cells (Fig 10B).

## Discussion

*P. vortex *is a fascinating organism, one with social motility that is exquisitely sensitive to its environment. Previously studies have concentrated on the mathematics of pattern formation, analyzing slowly developing colonies over a period of days and controlling the quality of the agar substrate precisely to ensure reproducible macroscopic morphologies [16]. Here we have taken a different approach, one that focuses on imaging and tracking at the cellular level. The choice of rich medium and low percentage agars suited real-time observation. High percentage agars permit the observation of greater structure whilst lower percentages permitted longer term imaging without inhibition of motility, possibly due to a greater resistance to desiccation.

This is the first detailed description of *P. vortex *swarming at the microscopic level. A picture emerges in which curved cells align into rotating or refluxing masses with what appears to be rapidly reversible physical coupling and coordination. The experiments in which a RIF^R ^strain was seeded into RIF^S ^colonies allowed tracking of the dispersal of cells within colonies. These experiments indicated that *P. vortex *was able to move within disparate locations with little bias, suggesting that the complex patterns formed are single colonies with the potential for communication and spread of cells, across several centimeters. Light microscopy of swarming *P. vortex *cells stained for flagella indicated that swarming cells had multiple flagella. SEM imaging detected filaments, consistent with the staining experiments and situations in which motility was observed, and therefore suggesting these were the flagella. The filaments viewed by SEM did not form helical bundles but rather tend to entangle or otherwise associate with those of neighboring cells. In this respect *P. vortex *resembles the swarming form of Aeromonas [60] for which lateral, entangling flagella are important in rafting. The loose nature of the connection suggested by the dispersion and reformation kinetics of swarming *P. vortex *(with dilution and the movement of clumps of bacteria within larger masses) is consistent with this idea. These observations suggest a model for the swarming of *P. vortex *that involves reversible connections formed by flagella and that a raft of curved cells can convert the rotation of individual flagella into the translocation of the entire colony. The loose nature of the contacts could allow a dual role for flagella in motility and rafting, maintaining the cohesion of the mass but permitting a degree of rearrangement including the characteristic vortex motion. Preliminary results from the genome sequencing of *P. vortex *suggest there is only one flagellar motility system. It is likely that flagella ids cohesion and drives motility but we cannot exclude the involvement of other mechanisms.

When cellular masses do part on higher percentage agar, it is the main body of cells that withdraws. Cell masses can meet but there is at least a short-term aversion to this event (Fig. 2D, Fig. 3D) that may promote efficient colonization of a surface without complete depletion of nutrients and therefore facilitate sporulation. In the longer term, we note that merging of multiple colonies on high-nutrient media did occur but that during growth on more limited nutrients there is a significant aversion to colonies merging as previously described. It is not clear if physical or sensory (e.g. negative chemotaxis) or other factors govern these interactions. Experiments with cell extracts revealed detergent activity that might be due to a surfactant such as surfactin, which is important in *B. subtilis *swarming. The dispersal effect (Fig. 4) seen in response to contact with a cell extract is consistent with this possibility. Preliminary genome sequencing data [66] are also consistent with the possibility of surfactin synthesis.

Within the mass of cells there may be distinct subpopulations. Morphological diversity was certainly commonplace within swarming cultures. Extremely elongated cells were a small but consistent fraction of the swarming population. Elongated cells were not hyperflagellated, and therefore do not resemble the swarmers of *Proteus mirabilis*, which have an unusually high density of flagella compared to the vegetative form [51]. We also note that under stress (Congo Red, MitC, elevated temperature) even more extreme and diverse morphologies (e.g. cells elongated and curved to the point of being twisted) emerged. The most informative stress was MitC. Preliminary sequencing data [66] and the visible filamentation suggested that this compound was acting as a trigger of the SOS response [64,65]. Sublethal doses of MitC resulted in less coordinated swarming, allowing more detailed observation of small cell masses with extreme morphologies. The MitC experiments, along with dilution of untreated motile cells, suggested that motility was a collective phenomenon; it was possible but never rapid in isolated cells. Additionally, a small number of cells (< 10) were sufficient to trigger the rapid rotational "vortex" movement that appears to be at the heart of the *P. vortex *pattern formation. In this situation, motile cells were usually in contact with non-motile cells and multiple foci of rotation could also be in contact (e.g. Fig. 9). Single cells displayed limited shunting or rotational movement but it was never as rapid as seen in collectives of cells, suggesting a genuinely collective or social behavior.

Two compounds were identified which limited swarming. One, Congo Red, is known to modulate a wide variety of motile behavior in bacteria, including gliding and flagella-mediated swarming [27]. Congo Red has multiple effects on cell surfaces and can interfere with gliding and flagellar motility [67]. Growth of *P. vortex* with Congo Red led to loss of motility and increased growth within the center of the inoculation and a subpopulation cells with extreme twists. Previous workers have suggested that the formation of a ring structure by *B. subtilis *is independent of motility [68]. For *P. vortex *this appears only partially true; a ring colony (on high percentage agars, Fig 10C) certainly does form in the absence of motility. But swarming appears to play a role in evacuating the center of the colony as well as outgrowth from the outer edge of the inoculation. A second inhibitor of *P. vortex *swarming, PNPG, is also a retardant of *Proteus mirabilis *swarming and is effective against *P. vortex *at a similar concentration (0.5 mM). This may imply parallels between these two phylogenetically quite distinct organisms. Both PNPG and Congo Red have a practical utility in facilitating conventional single colony microbiology of *P. vortex*. Additionally, mutants that overcome these inhibitors are likely to be valuable in understanding the mechanism of swarming.

Finally, there is the question as to the practical applications of pattern formation in bacteria beyond being a model system for processes such as complex cellular organization. One possibility is in the patterning or construction of microscale devices where the structure must be constructed according to flexible rules rather than an exact template [69]. Secondly, *P. vortex *is exquisitely sensitive to changes in its environment and alters its pattern formation visibly with very subtle environmental changes. This might be an interesting method for monitoring environments that must be kept extremely consistent. Thirdly, we note that complex pattern formation is a property rapidly lost with laboratory culture but is one sometimes found in recently isolated pathogens including *Paenibacillus alvei *[16,56]. We speculate that it may therefore be of advantage in infections, as quorum sensing has already been found to be [40,41]. Antimicrobials targeted against complex social behavior, i.e. quorum sensing, are already in development. *Paenibacillus *as a model system may allow new targets limiting "antisocial" behavior to be identified.

## Conclusion

*P. vortex *in a bacterium which swarms on nutrient agars to form intricate, extended colonies, within which individual cells have considerable ability to relocate. Cell morphology, vigorous motility (apparently driven by flagella) and interactions between moving masses of cells are important factors in the dynamic architecture of these complex colonies. This work represents an in-depth examination of bacterial cooperative organization from the cell up to the colony level. The results may give important clues about bacterial strategies of cooperative adaptability to environmental stresses. *P. vortex *provides a valuable model for biocomplexity and the self-organization of biological systems in general.

## Methods

### Culture

Liquid culture of *P. vortex *was in Mueller Hinton (MH, from Oxoid, UK) broth at 37°C with vigorous shaking. Growth in liquid culture was measured as OD_595 _using a Hitachi U-1500 spectrophotometer. For swarming, MH agar plates (see Results for agar concentrations) were poured with 20 ml of medium and were allowed to solidify whilst covered with sterile filter paper to ensure a consistent thickness and unblemished surface without air bubbles or drying patterns. Congo Red, Mitomycin C and PNPG (all Sigma, NL) were dissolved in distilled water, filter sterilized and added to liquid growth medium, or to MH agar just before pouring, at the appropriate concentrations. Inoculations for swarming were made by depositing a 10 μl drop of an overnight culture grown in MH broth onto the center of a MH agar plate and incubating for 5–60 h at 37°C. Multiple inoculations were made on the same plate to look at the interactions between colonies. Cells were not subcultured indefinitely, every two weeks a new culture was grown from stocks stored at -80°C.

### Light microscopy and imaging of motility

Organisms swarming on the surface of nutrient agars were imaged by transmission light microscopy using an Olympus BX41 microscope with a total magnification of 40–500 fold (Olympus, Japan). A heated microscope stage (Marzhauser, Germany) was used to incubate plates during real-time imaging. Image capture was by Kappa CCD camera. Image analysis and the compilation of stacks of still images into movies was with ImageJ software [70]. Addition of compounds to swarming cells was performed manually with a sterile toothpick targeted by low-power microscopy. A Leica DSZ binocular microscope (Leica, NL) with an intrinsic digital camera was used for low magnification (8 to 40 fold) imaging.

### Scanning electron microscopy

Organisms actively swarming on the surface of MH agar were rapidly fixed *in situ *for imaging by scanning electron microscopy (SEM). This was done by excising a 3 × 3 mm area of interest with a razor blade, placing the fragment in an empty Petri dish and surrounding the agar fragment on all sides (except the upper surface) with MH broth containing 3 % (v/v) glutaraldehyde (Fluka, NL). After 2 h fixation, followed by three washes in MH broth, the samples were incubated for 15 min in 1 % (w/v) osmium tetroxide to preserve extracellular polymers. Dehydration with ethanol, critical point drying and imaging were performed as previously described [71]. Additionally, a paraformaldehyde/glutaraldehyde vapour fixation method was used for as a control for fixation artifacts [61].

### Preparation of extracellular material from swarming cells

Inoculations of *P. vortex *were made onto MH agar (1.5 % w/v) plates as above then: (a) 12 plates with swarms from 6 to 8 cm in diameter were incubated for 14 h, after which all areas of microbial growth (viewed by microscopy) were highly motile and (b) 12 plates were incubated for 60 h, after which the swarm had reached the edges of the Petri dish and motility had completely ceased. Preparations of extracellular material were made from both original sets of Petri dishes by harvesting the cells in 1 ml of MH broth per plate and pooling preparations made under the same condition. Harvested material was centrifuged twice to clear the cells (4500 g); the supernatant was filtered through a 0.22 μm filter and stored at -80°C in individual aliquots. A mock harvesting was made from 12 MH plates without inoculation as a control. Filtered medium from overnight (o/n) liquid cultures was also prepared. The resulting cell-fee supernatants were tested in three ways: (a) 1% (v/v) of the preparation was added to 1.5% (w/v) agar MH plates, then 10 μl of an o/n *P. vortex *culture was inoculated in the center and the plates were incubated at 37°C; (b) a *P. vortex *o/n culture was pelleted by centrifugation (4500 g) and resuspended in an identical volume of supernatant, then 10 μl aliquots were inoculated onto 1.5% agar (w/v) MH plates which were incubated at 37°C; (c) detergent activity was determined, as described below.

### Detergent activity assays

The detergent activity of extracts was tested using a drop spreading method [72]. Briefly, this was done by spotting 15 μl droplets of water containing 1% (v/v) toluidine blue onto 1 mm thick films of 1 % (w/v) low melting point agarose (Invitrogen, NL) and observing the spreading properties. The agarose contained extracellular material from swarming cells (10% or 50% v/v) or 10^8 ^cfu of cells (after washing twice in MH medium), or as controls MH medium or detergents at known concentrations.

### Tracking of the dispersal of antibiotic-resistant mutants within colonies

A spontaneous rifampicin resistant mutant of *P. vortex *was isolated from the parental strain in a similar fashion to mutants selected for in *B. subtilis *[62]. MH agar (1.5% w/v) containing 50 μg/ml rifampicin (Sigma, NL) was used to select rifampicin-resistant (RIF^R^) mutants. One such RIF^R ^mutant was selected and used to track the dispersal of this variant through a wild-type colony. Macroscopic colonies of wild-type (RIF^S^) *P. vortex *were inoculated with approximately 10^4 ^cfu of motile Rif^R ^mutant from another MH plate using a sterile toothpick. For each experiment a single inoculation point was chosen for the RIF^R ^strain at different locations and stage of development of the wild-type colony. After 1–20 h further incubation at 37°C the pattern on the plate was replicated using a sterile velvet pad onto fresh MH with and without RIF (50 μg/ml) and cultured for 16 h at 42°C to limit swarming. The pattern of growth on the replica RIF plate was used to assess the degree to which the mutant strain penetrated the network of the wild-type colony.

### Flagella staining

Staining of flagella was preceded by gentle transfer of swarming cells into drops of water or into warmed MH medium on a microscope slide using a sterile toothpick. Staining was by the tannic acid/crystal violet protocol of Heimbrook and coworkers [73] with examination of flagella by transmission light microscopy.

## Authors' contributions

CI performed and devised "wet lab" laboratory experiments and with EBJ wrote the manuscript. Sequence analysis, bioinformatics, development of procedures for handling *P. vortex *and isolation of the organism and speciation were provided by EBJ. Both authors have approved the final manuscript.
